# Predictive factors for functional failure of ventral mesh rectopexy in the treatment of rectal prolapse and obstructed defecation

**DOI:** 10.1007/s10151-022-02708-8

**Published:** 2022-10-04

**Authors:** S. Solari, J. Martellucci, S. Ascanelli, A. Sturiale, A. Annicchiarico, B. Fabiani, P. Prosperi, P. Carcoforo, G. Naldini

**Affiliations:** 1 Department of Medical Science, University of Ferrara, Ferrara, Italy; 2 Emergency Surgery, Careggi University Hospital, Florence, Italy; 3 Proctology and Pelvic Floor Clinical Center, University Hospital of Pisa, Pisa, Italy; 4 Department of Medicine and Surgery, University of Parma, Parma, Italy

**Keywords:** Rectal prolapse, Constipation, ODS, Ventral mesh rectopexy, Proctology, Pelvic floor

## Abstract

**Background:**

Ventral mesh rectopexy (VMR) is widely accepted for the treatment of rectal prolapse or obstructed defecation. However, despite good anatomical results, the improvement of functional symptoms (constipation or incontinence) cannot always be obtained and in some cases these symptoms may even worsen. The aim of the present study was to identify possible predictors of functional failure after VMR.

**Methods:**

Data of all consecutive patients who had VMR for the treatment of rectal prolapse and/or obstructed defecation between January 2017 and December 2020 in three different pelvic floor surgical centres in Italy were analysed to identify possible predictors of functional failure, intended as persistence, worsening or new onset of constipation or faecal incontinence. Symptom severity was assessed pre- and postoperatively with the Wexner Constipation score and Obstructed Defecation Syndrome score. Quality of life was assessed, also before and after treatment, with the Patients Assessment of Constipation Quality of Life questionnaire, the Pelvic Floor Disability Index and the Pelvic Floor Impact Questionnaire. Faecal incontinence was evaluated with the Cleveland Clinic Incontinence Score. The functional outcomes before and after surgery were compared.

**Results:**

Sixty-one patients were included (M:F ratio 3:60, median age 64 years [range 33–88 years]). Forty-two patients (68.9%) had obstructed defecation syndrome, 12(19.7%) had faecal incontinence and 7 patients (11.5%) had both. A statistically significant reduction between pre- and postoperative Obstructed Defecation Syndrome and Wexner scores was reported (*p* < 0.0001 in both cases). However, the postoperative presence of constipation occurred in 22 patients (36.1%) (this included 3 cases of new-onset constipation). The presence of redundant colon and the pre-existent constipation were associated with an increased risk of persistence of constipation postoperatively or new-onset constipation (*p* = 0.004 and *p* < 0.0001, respectively). The use of postoperative pelvic floor rehabilitation (*p* = 0.034) may reduce the risk of postoperative constipation.

**Conclusions:**

VMR is a safe and effective intervention for correcting the anatomical defect of rectal prolapse. The degree of prolapse, the presence of dolichocolon and pre-existing constipation are risk factors for the persistence or new onset of postoperative constipation. Postoperative rehabilitation treatment may reduce this risk.

## Introduction

Faecal incontinence is reported in 50–88% of patients with rectal prolapse. Mucus leakage is often described early in the disease process and may evolve into frank faecal leakage [1]. Constipation is reported in 25–70% of patients with rectal prolapse and may be the result of prolapsed bowel leading to obstruction, although the causal relationship is unclear [2].

Ventral mesh rectopexy (VMR) was first developed for the treatment of prolapse and constipation and became wildly accepted in clinical practice [3]. Compared to conventional open surgery, laparoscopic VMR has many advantages: a smaller wound, shorter recovery time, low morbidity and an overall improvement in continence [4], but long-term results are still lacking and the data currently available in the literature do not permit identification of a surgical gold standard.

Comparative studies between the various rectopexy techniques are still inconclusive: which is the best option for the mobilisation of the rectum, the choice of prosthetic material and the site of fixation, or whether and when the addition of sigmoid resection may be useful remain undecided. [1, 5]. Despite a recurrence rate, between 2.5 and 14%, the rate of improvement of symptoms does not exceed 76% for constipation and 62% for faecal incontinence [6]. New-onset constipation can develop in up to 30% of cases [4]. However, it is still unclear which patients are most at risk of failure. Only a few studies have researched risk factors for failure, and results have been heterogeneous [7–9].

Identification of patient populations at high risk of failure might allow a targeted surgical strategy based on the characteristics of the patient, prolapse and symptoms.

In the present study, we analysed a multicentre case series of patients who had VMR, either laparoscopic or robotic, for rectal prolapse and obstructed defecation. Our aim was to identify possible predictors of failure, i.e. of persistence, worsening or new onset of constipation.

## Materials and methods

In this multicentre study, we evaluated consecutive patients who had VMR for the treatment of rectal prolapse and/or obstructed defecation associated with anatomical alterations (internal or external prolapse, rectocele), between 1 January 2017 and 31 December 2020, in three different surgical centres: the Careggi University Hospital of Florence, the S. Anna University Hospital of Ferrara and the Cisanello University Hospital of Pisa. Data were prospectively collected in a dedicated database and retrospectively analysed. Written informed consent was obtained from all patients. Ethical approval was obtained from the local Ethics Committees.

Inclusion criteria were patients over 18 years old diagnosed with external rectal prolapse or internal rectal prolapse with obstructed defecation syndrome (ODS) associated with anatomical anomalies such as rectocele, descending perineum, enterocele and multicompartmental prolapse of pelvic organs (POP). All patients failed conservative behavioural and medical treatment.

Exclusion criteria were surgical treatment of prolapse with perineal approach, open surgery or conversion to open, chronic pelvic pain, presence of multicompartmental prolapse requiring combined operations, malignancy, inflammatory bowel disease, megacolon, pregnancy and contraindications to general anaesthesia.

The primary endpoint was possible predictors of functional failure, i.e. of persistence, worsening or new onset of constipation.

Data collected included patient demographics, medical history, preoperative diagnostic work-up, including physical examination, anorectal manometry, defecography or dynamic pelvic magnetic resonance imaging (MRI), which is routinely performed in normal clinical practice in these hospitals. Surgical data collected were operating time, length of hospital stay, complications and reoperations. Follow-up data were collected during clinical evaluation performed at 1, 6 and 12 months postoperatively and then every year until October 2021. These data included improvement or persistence of symptoms, quality of life, patient satisfaction and recurrence. Symptom severity was assessed pre- and postoperatively with the Wexner Constipation score (WCS) [10] and ODS scores [11]. Quality of life was assessed, before and after treatment, with the Patients Assessment of Constipation Quality of Life (PAC-QoL) [12], the Pelvic Floor Disability Index (PFDI) [13], and Pelvic Floor Impact Questionnaire (PFIQ) [14]. Faecal incontinence was evaluated with the Cleveland Clinic Incontinence Score, (CCIS) [15]. Defecatory function was compared before and after surgery for each patient. Functional failure of surgical treatment was defined as the persistence or new onset of constipation, defined by a WCS > 15. Rectal prolapse was defined according to the Oxford classification [16].

Surgery was performed with laparoscopic or robotic access. During ventral rectopexy the anterior plane of the rectum was dissected from the vagina downwards to the levator plane. A strip of rectangular titanized polypropylene mesh (TiLOOP, SunMedical, Segrate, Italy) or porcine dermal collagen implant (Permacol, Medtronic, Minneapolis, USA) was placed at the lowest point of the rectovaginal space and attached to the anterior wall of the distal rectum. The proximal end of the prosthesis was sutured to the sacral promontory after upward traction of the rectum. The peritoneal incision was then closed to cover the prosthesis and obliterate the cul-de-sac.

We analysed the data and identified the patients with no improvement or worsening of the symptoms, looking for possible correspondences of the preoperative characteristics of the patients, such as age, comorbidities, body mass index (BMI), and pre-existing constipation (defined as functional constipation according to the Rome IV criteria without prevalent symptoms of ODS such as straining, anorectal blockage, incomplete evacuation, manual manoeuvres), faecal incontinence, preoperative rehabilitation, postoperative rehabilitation, presence of a redundant colon (dolichocolon) verified by a preoperative colonoscopy and confirmed during surgery, previous pelvic surgery or obstetric trauma, type of prosthesis used, robotic/laparoscopic approach.

### Statistical analysis

We analysed data using the Statistical Package for the Social Sciences software (SPSS Inc., Chicago, IL, USA).

The *χ*2 test was used to compare categorical variables when appropriate. The Mann–Whitney *U* test was used for continuous, non-normally distributed variables and an independent-sample *t* test was used for continuous, normally distributed data. Data were expressed as mean ± SD or median (interquartile range). *p* < 0.05 was considered statistically significant.

## Results

### Patient characteristics

Demographic data, history, prolapse features and symptoms are summarised in Table 1. Sixty-one consecutive patients who underwent surgery for external rectal prolapse (12 patients) and ODS during the study period were included (60 females [98.4%], median age 64 years [range 33–88 years] at the time of surgery). Rectocele > grade II was found in 42 patients. Thirty-five patients (57.4%) had a history of vaginal delivery (average 1.8 pregnancies per patient) 25 of whom had reported obstetric perineal trauma. 30 (49.2%) had a history of previous pelvic or perineal surgery: hysterectomy (*n* = 19), urinary incontinence surgery (*n* = 4) stapled transanal rectal resection (STARR) (*n* = 3), hemorrhoidectomy (*n* = 2), Altemeier perineal proctosigmoidectomy (*n* = 2), fistulectomy (*n* = 1).

**Table 1:** Patients’ demographics at presentation

Total number	61
Females, *n* (%)	60 (98.4)
Age (years), median (range)	64 (33–88)
ASA class, mean ± SD	2.33 ± 0.5
BMI (kg/m^2^), mean ± SD	25.8 ± 4.3
Vaginal deliveries, mean ± SD	1.8 ± 0.9
Obstetric trauma, *n *(%)	25 (41)
Previous pelvic surgery, *n* (%)	30 (49.2)
Menopause, *n* (%)	50 (82)
Hormonal therapy, *n* (%)	6 (9.8)
Sexually active, *n* (%)	27 (44.3)
External rectal prolapse, *n* (%)	12 (20)
Descending perineum, *n* (%)	38 (62.3)
Redundant colon, *n* (%)	17 (27.9)
ODS (ODS score > 10), *n* (%)	42 (68.9)
Preoperative faecal incontinence, *n* (%)	12 (19.7)
ODS and incontinence, *n* (%)	7 (11.5)
Pre-existing constipation (Wexner score > 15), *n* (%)	22 (36.1)
Urinary stress incontinence, *n* (%)	19 (31.1)
Preoperative rehabilitation, *n* (%)	14 (23)

*SD* standard deviation, *ASA* American Society of Anesthesiologists, *BMI* body mass index, *POP* pelvic organ prolapse, *ODS* obstructed defecation syndrome

Forty-two patients (68.9%) presented with ODS, 12 (19.7%) had severe or complete faecal incontinence (CCIScore > 15), while 7 patients (11.5%) had both ODS and incontinence. Redundant colon was present in 17 patients (27.9%). Fourteen patients (23%) had already failed rehabilitation therapy.

Patients underwent ventral rectopexy surgery with laparoscopic (54.1%) or robotic (45.9%) access, with placement of a biological implant (Permacol) in 35 cases (57.4%) and titanized synthetic polypropylene mesh (TiLOOP)) in the others.

The mean operating time was 135 ± 46.5 min with a mean length of hospital stay of 3.8 ± 1.3 days and a complication rate of 18%, almost all of them within Clavien-Dindo grade II. Only one patient required surgery for a grade IIIb complication (incisional hernia repair). A total of five patients presented with anatomical internal rectal prolapse recurrence (residual intussusceptions) two of whom had the initial surgery for rectal prolapse and three for ODS. Surgery details and complications are reported in Table 2.

**Table 2:** Clinical results

Operating time (minutes), mean ± SD	135.9 ± 46.5
Robotic surgery *n* (%)	28 (45.9%)
Biologic prosthesis *n* (%)	35 (57.4)
Length of hospital stay (days), mean ± SD	3.8 ± 1.3
Complications *n* (%)	11 (18)
Suture hematoma	1
Wound dehiscence	1
Urinary infection	1
Presacral hematoma	1
Wound infection	3
Incisional hernia	3
Reoperation for complications	1
Length of follow-up, months, mean ± SD	27.7 ± 8.49
Sexual life improvement/27 (%)	22 (81.5)
Prolapse recurrence, *n* (%)	5 (8.2)
Constipation recurrence, *n* (%)	19 (31.2)
New-onset constipation, *n* (%)	3 (4.9)
Interval between surgery and recurrence, months, mean ± SD	21.1 ± 8.47
Reoperation for recurrence, *n*	3

*SD* standard deviation

The mean duration of follow-up was 27.7 months (range 6–60 months).

Functional scores results are reported in Table 3. In almost all cases (except for PAC-QoL B7) there was a statistically significant reduction between pre- and postoperative scores, with an improvement in all items. Out of 27 sexually active patients, 22 (81.5%) reported an improvement in sex life after surgery.

**Table 3:** Pre- and postoperative functional results

	Preoperative	Postoperative	*p* value
ODS score (mean ± SD)	18.9 ± 7.1	8.4 ± 0.7	< 0.0001
Wexner score (mean ± SD)	18.7 ± 3.5	10.7 ± 2.8	< 0.0001
PAC-QoL B1–B6 (mean ± SD)	62.7 ± 24.5	36 ± 3.5	< 0.0001
PAC-QoL B7 (mean ± SD)	5.2 ± 1.4	6.4 ± 5.7	0.0878
PFDI score (mean ± SD)	152.2 ± 28.2	65.4 ± 20.3	< 0.0001
PFIQ score (mean ± SD)	147.1 ± 75.1	56 ± 10.4	< 0.0001

*ODS* obstructed defecation syndrome, *PAC-QoL* patient assessment of constipation-quality of life, *PFDI* pelvic floor disability index, *PFIQ* pelvic floor impact questionnaire, SD standard deviation

Postoperative constipation, defined by a WCS > 15, was recorded in 22 patients (36.1%) 3 of whom with new onset, on average 21 months after surgery. Faecal incontinence (defined as CCIS > 15) was significantly reduced postoperatively (*p* = 0.0011). However persistence of faecal incontinence was reported in five patients (all treated for external rectal prolapse) and new-onset incontinence in one.

Characteristics of patients with persistent or new-onset constipation (group A, *n* = 22) were compared with those of patients without postoperative constipation (group B, *n* = 39) and reported in Table 4. Age, BMI, American Society of Anesthesiologists (ASA) class, history of obstetric perineal injuries or pelvic-perineal surgery did not seem to significantly influence the surgical outcome. Similarly, we found no significant differences according to the type of prosthesis used (biological or synthetic) and the type of surgical approach (laparoscopic or robotic). On the other hand, the presence of a redundant colon and the presence of pre-existing constipation were associated with a higher risk of persistence or new onset of constipation in the postoperative period (*p* = 0.004 and *p* < 0.0001, respectively). Postoperative pelvic floor rehabilitation treatment (*p* = 0.034) may reduce the risk of postoperative constipation.

**Table 4:** Risk factors for failure

	Group A	Group B	*p* value
Number of patients	22	39	
Age (years), median (range)	68.5 (46–80)	64 (33–88)	0.8
ASA class, mean ± SD	2.3 ± 0.5	2.3 ± 0.6	0.917
BMI (kg/m^2^), mean ± SD	25.5 ± 4.5	26 ± 4.3	0.767
Obstetric trauma, *n* (%)	10 (45.5)	15 (38.5)	0.601
Previous pelvic surgery, *n* (%)	11 (50)	19 (48.7)	1
Redundant colon, *n* (%)	12 (54.5)	7 (18)	**0.004**
Pre-existing constipation, *n* (%)	16 (72.7)	9 (23.1)	**< 0.0001**
Preoperative rehabilitation, *n* (%)	7 (31.8)	7 (16.7)	0.342
Postoperative rehabilitation, *n* (%)	7 (31.8)	24 (61.5)	**0.034**
Robotic surgery, *n* (%)	10 (45.5)	18 (46.2)	1
Biologic prosthesis, *n* (%)	13 (59.1)	22 (56.4)	1

Statistically significant *p* values (*p* < 0.05)

*Group A* patients with persistent or new-onset postoperative constipation, *Group B* patients without postoperative constipation, *BMI* body mass index, *ASA* American Society of Anesthesiologists, *SD* standard deviation

## Discussion

VMR is currently adopted by many surgeons as the procedure of choice for the treatment of internal and external rectal prolapse as well as symptomatic rectocele. The laparoscopic approach proved to be non-inferior to a laparotomic access in terms of functional outcomes and recurrence rates and may be superior in terms of morbidity [17, 18]. The incidence of prosthesis erosion is low and is more common after synthetic mesh placement [19]. Even in our study, the type of access, laparoscopic or robotic and the type of prosthesis used did not affect the functional results of rectopexy surgery.

In 24% of the cases, a preoperative rehabilitation treatment was attempted, without success. Rehabilitation is not currently recommended in clinical practice for the treatment of rectal prolapse [1, 5]. There are few published studies on the rehabilitative treatment of rectal prolapse, which suggest that it plays no role in the treatment of prolapse particularly in the presence of relevant anatomical abnormalities, either when constipation is present or when there is faecal incontinence [20]. Our study confirms this finding, since 23% of patients had had preoperative rehabilitation treatment, but still needed surgery. Moreover, there were no differences in the rate of preoperative rehabilitation between patients with effective surgical treatment and those with ineffective surgical treatment. In some patients, postoperative rehabilitation treatment was also performed when clinical and instrumental examination suggested the presence of a concomitant functional disorder of the pelvic floor as the cause of constipation. Interestingly, we observed that the effectiveness of postoperative rehabilitation treatment in reducing the risk of persistence or new onset of constipation after VMR (*p* = 0.034, Table 4). We hypothesised that in these patients rehabilitation may not effectively confer its intended benefit while the anatomical defect is still present. Once prolapse is corrected, the pelvic floor and sphincter muscles are relieved and might start to regain their normal function. Postoperative rehabilitation could be routinely applied to reduce the risk of persistent constipation.

The patients we analysed showed a significant improvement in defecation with a significant improvement in all the scores examined, except for the last item of the PAC-QoL questionnaire. The latter investigates the degree of patient satisfaction related to constipation and shows a slight improvement between pre- and postoperative, although it is not statistically significant (*p* = 0.0878). This finding, in contrast to the other scores, shows the possible gap between success measured with the specific score items and the patient's perception of the impact of the treatment on their overall quality of life.

Postoperative constipation was observed in 22 patients (36.1%), among which 3 (4.9%) had new-onset constipation, a slightly lower incidence rate than that in a review of different rectopexy techniques, which reported rates of new-onset constipation ranging from 5.5 to 10.55% for VMR [21].

Our study showed a reduction, although not significant, in faecal incontinence after VMR (*p* = 0.2152). On further analysis, we found a reduction in incontinence in patients who complained of it preoperatively, but we also recorded some cases of new-onset incontinence. This agrees with the results of previous studies [22, 23]. The improvement of continence can be explained by the elimination of intussusception, which caused inappropriate activation of the recto-anal inhibitory reflex, or by a reduction in incomplete rectal emptying [24]. Patients who did not improve after surgery may have had other underlying factors that caused faecal incontinence, such as anal sphincter insufficiency or neurological factors [25].

There are few studies in the literature that attempt to identify the causes of anatomical or functional failure of ventral rectopexy. A 2017 study analysed the recurrence rates of rectal prolapse after LVR surgery, focusing on the anatomical recurrence of prolapse. As in our study, Fu et al. analysed possible predictors of recurrence, highlighting preoperative alteration of pudendal nerve motor latency and use of synthetic prosthesis as significantly associated. Age, previous prolapse surgery, incontinence and preoperative manometric parameters were not predictive of recurrence [7]. Similarly, in a 2019 meta-analysis of 17 studies and 1242 patients, Emile highlighted male sex and prosthesis length of less than 20 cm as factors significantly associated with anatomical recurrence of full-thickness prolapse. Age, BMI, previous prolapse surgery, type of prosthesis used, duration of surgery, number of surgeons and conversion rate to open surgery were not predictive [8]. Only one published study examines surgical failure from the perspective of persistence of ODS symptoms [9]. The study showed that obstetric trauma and total number of deliveries do not influence the outcome in terms of constipation after VMR, whereas a higher BMI leads to an increased risk of anatomical and functional recurrence.

We also focused on recurrence in a clinical and functional sense, i.e. as persistence of constipation symptoms that brought the patient to the surgeon’s attention, or as new onset of symptoms. We tried to expand the range of possible risk factors analysed (Table 4). Compared to the studies mentioned above, the type of the prosthesis, male sex and BMI did not influence the clinical success of VMR for constipation in our case series. Consistent with the findings of Kremel et al., previous obstetric trauma and the number of deliveries did not influence the clinical outcome.

The most important parameter that is associated with an increased risk of clinical recurrence is the higher degree of prolapse according to the Oxford classification. In this case, however, the finding is to be correlated with an increased risk of prolapse recurrence, especially in major external prolapse, with a directly proportional relationship between prolapse extent (in centimetres) and risk of recurrence. It seems possible that the persistence of constipation in these patients is concomitant with recurrence or incomplete treatment of the underlying anatomical problem. On the other hand, the impact of prolonged and severe constipation due to multifactorial causes cannot be ignored, and the onset of prolapse and the worsening of its clinical manifestation that could be related as cause/effect.

Similarly, the presence of a redundant colon has been shown to be a risk factor for recurrence. Redundancy of the sigmoid colon was found on preoperative colonoscopy or intraoperatively. This is not surprising: according to guidelines, sigmoid resection can be added to posterior rectopexy in patients with prolapse and constipation [5]. Resection-rectopexy is a safe and effective procedure that achieves a better outcome in cases of constipation, especially in patients with a redundant sigma and a symptomatic sigmoidocele [26]. In 1992, a study showed that patients undergoing rectopexy alone had a higher pressure in the rectum for a given volume of isotonic sodium chloride solution introduced [27]. The authors hypothesised that this was due to a kinking between the redundant sigmoid and the rectum at the rectosigmoid junction. The addition of sigmoid resection could reduce this problem by avoiding the kinking that could be the cause of delayed passage of bowel contents. However, some studies have shown that although the functional results of resection-rectopexy are similar to those of VMR, but that postoperative complications might be greater [28–31]. The extent of colonic resection, method of mobilisation and rectal fixation vary considerably in the literature. Colectomy is usually not recommended in combination with repairs involving a prosthesis. However, some papers reported good functional results for resection-rectopexy procedures with ventral [32] or dorsal prosthesis placement [33, 34], albeit with higher morbidity rates. However, a procedure conducted with minimal contamination, irrigation of the pelvic surgical area, complete closure of the pelvic peritoneum and the use of a dorsally placed biologic prosthesis may reduce the risk of potential pelvic infections and complications.

The presence of constipation prior to prolapse also increases the risk of clinical failure. This finding confirms the multifactorial nature of constipation and ODS: even after correction of the anatomical defect, intestinal motility, hormone levels, psychological aspects and other factors can influence the patient's defecatory function. A 2014 study suggested that a significant number of patients presenting with rectal prolapse had an altered colonic transit time. Despite extensive resections of the entire left colon, the altered colonic transit could not be corrected, while some patients developed new-onset constipation [35]. In a defecographic study of rectal motility, obstructed defecation persisted after rectopexy, apparently due to the fixity of the rectum preventing effective expulsive contraction [36].

This study has several limitations: the retrospective and multicentric design, that did not allow standardization of follow-up, the limited number of patients, the absence of control groups and the involvement of both external rectal prolapse and ODS with internal rectal prolapse.

The treatment of constipation requires a multidisciplinary approach for a long period after surgery: about 20% of patients with ODS and 25% of patients suffering from anal incontinence have persistent symptoms and still seek help [37]. Currently, there is no evidence indicating what the optimal treatment after failure of VMR might be, but it is mandatory to study accurately the patient and their clinical presentation before choosing the treatment.

## Conclusions

Prosthetic ventral rectopexy is a safe and effective intervention to correct the anatomical defect of external rectal prolapse and anatomical alterations related to ODS. Functional results, however, may not always be optimal, especially in terms of persistence or new onset of constipation. The presence of dolichocolon and long-term constipation are risk factors for the persistence or new onset of postoperative constipation. Postoperative rehabilitation treatment may reduce this risk. A thorough study of the patient's characteristics, symptoms and anatomical alterations is essential in planning the best treatment strategy.

## Data Availability

The datasets analysed during the current study are available from the corresponding author on reasonable request.
